# Effects of *Saccharomyces cerevisiae* and *Kluyveromyces marxianus* on the Physicochemical, Microbial, and Flavor Changes of Sauce Meat during Storage

**DOI:** 10.3390/foods13030396

**Published:** 2024-01-25

**Authors:** Lili Ji, Shu Wang, Yanan Zhou, Qing Nie, Chunyan Zhou, Jiawen Ning, Chunping Ren, Chun Tang, Jiamin Zhang

**Affiliations:** Meat Processing Key Lab of Sichuan Province, College of Food and Biological Engineering, Chengdu University, Chengdu 610106, China; lily_jee@126.com (L.J.); willywonka19@163.com (S.W.); zyn6089@163.com (Y.Z.); 13281811538@163.com (Q.N.); zhousprings@163.com (C.Z.); qq1061381916@163.com (J.N.); hahadan2868@126.com (C.R.); sctangchun@126.com (C.T.)

**Keywords:** *Saccharomyces cerevisiae*, *Kluyveromyces marxianus*, sauced meat, physicochemical properties, microbiological properties, flavor components

## Abstract

*Saccharomyces cerevisiae* (*S. cerevisiae*) and *Kluyveromyces marxianus* (*K. marxianus*) are often used as fermenters in yogurt and alcohol, and have been less studied within meat products. The yeasts were added to sauce meat, and the uninoculated group served as a control in this study to examine and compare the changing patterns of physicochemical and flavor characteristics of *S. cerevisiae* and *K. marxianus* on sauce meat during storage. The changes in moisture content, aw, pH, thiobarbituric acid reactive substances (TBARS), and other flavor characteristics were measured in sauce meat during the first, second, fourth, and sixth months after production. The following factors were examined: moisture content, aw, pH, TBARS, peroxide value (POV), acid value (AV), soluble protein (SP), free amino acid (FAA), and volatile flavoring compounds. With VIP > 1 and *p* < 0.05 as the screening conditions, the partial least squares model (PLS-DA) was used to assess the distinctive flavor components in the sausages. The findings demonstrated that the three groups’ changes in sauce meat were comparable during the first two months of storage but differed significantly between the 4th and 6th months. The moisture content, water activity, and pH of the sauce meat decreased gradually with the storage time; TBARS, AV, and FAA increased significantly; SP decreased significantly from 2.61 to 1.72, while POV increased to 0.03 and then decreased to 0.02. The POV and TBARS values of the yeast-infected meat were substantially lower than those of the control group, and the POV and TBARS values of the meat inoculated with *S. cerevisiae* were particularly decreased (*p* < 0.05). The POV and TBARS values of SC (*S. cerevisiae* group) decreased by 49.09% and 40.15%, respectively, compared to CK (the control group) at the time of storage until June. The experimental group (KM: *K. marxianus* group) significantly increased the SP and FAA values of the sauce meat (*p* < 0.05) by 32.4% and 29.84% compared to the CK group, respectively. Esters and olefins as well as alcohols and esters were much greater in meat that had been supplemented with *S. cerevisiae* and *K. marxianus* than in meat from the control group. In conclusion, inoculating sauce meat with *S. cerevisiae* can significantly enhance the quality and flavor of sauce meat while it is being stored.

## 1. Introduction 

Traditional preserved meat products are known for their salty and fresh taste, unique flavor and firmness, and their dry and easy-to-store characteristics are especially suitable for production in the Sichuan region with high humidity, which makes up for the shortcomings of meat products that are difficult to be preserved for a long period of time. Sauced meat is a traditional cured meat product in Sichuan that resembles bacon in appearance. In reality, there are some differences in the production process and auxiliary materials, mainly that bacon and sauced meat are traditionally made by trimming and curing the pork or thigh meat and air-drying it. In the curing process, the sauce meat will have special seasonings added, such as mash, bean paste, sweet been sauce, and so on, to make the flavor more saucy. Under long-term storage conditions, sauced meat continues to impart flavor, but the challenging production environment and harmful bacterial infestations can result in an uneven quality of sauce meat during the natural fermentation process [1,2], which is likely to be severely constrained when promoting large-scale production. 

The main flora in the spontaneous fermentation of meat products are lactic acid bacteria (LAB) and staphylococci (CNC) [3,4]. Meanwhile, many microorganisms, including lactobacilli, staphylococci, molds, and yeasts, have been widely used as fermentation agents in meat products [5,6,7,8], to improve food safety [9], add special flavor [10], etc. Yeasts can obtain free amino acids from meat products due to their property of hydrolyzing fats and proteins. The color and scent of meat products can be influenced by the oxygen consumption characteristics, amino acid breakdown, and hydrolysis of lipids and proteins, which can lead to the generation of ethanol, acetaldehyde, or ethyl acetate, as well as a number of other volatile compounds [11], and yeast can grow at a lower pH than lactobacilli [12,13]. Therefore, it is necessary to combine physicochemical and taste research with their actual use in sauced meat in order to examine the quality impacts of yeast on the quality of sauced meats during storage. *Saccharomyces cerevisiae* (*S. cerevisiae*) is widely used in the production of fermented foods [14,15,16] (bread, buns, fish), beverages [17,18] (beer, wine), etc. [19,20]. In addition to promoting the hydrolysis of the pork proteins, the fermentation of dried pork by a mixture of *Lactobacillus plantarum* and *S. cerevisiae* results in a distinctly mellow flavor [21]. Additionally, *Kluyveromyces marxianus* (*K. marxianus*) is frequently added to foods like milk beer, yogurt, natto, bread, etc. [22,23,24,25]. Furthermore, *K. marxianus*, a superior strain utilized in industrial fermentation production, has the benefits of high safety and rapid development, particularly in dairy products. Gao [26] employed four strains of *K. marxianus*, five strains of *S. cerevisiae*, and six strains of *Pichia pastoris* (*P*. *pastoris*) to join in the pilot production of milk beer. It was ultimately confirmed that *K. marxianus* BJ1 was a high-grade milk yeast for milk beer and that its fermented milk beer was of the best quality by physicochemical and hygienic testing, genetic stability tests, organoleptic assessments, and volatile composition studies. Koo et al. [27] found that inoculation of three lab strains (*L. animalis*, *L. amylovorus*, and *P. acidilactici*) into frankfurter sausages had an inhibitory effect on *Listeria monocytogenes*, and the results showed that *Listeria monocytogenes* was inhibited after eight weeks of refrigeration under the same conditions compared to the control group without the additive. *L. monocytogenes* was reduced by 0.6 log after eight weeks of refrigeration under the same conditions compared to the control group without additives. The *S. cerevisiae* inoculated in fermented fish products by Liao et al. [28] mildly degraded Trimethylamine-N-oxide (TMAO) through the TMAO demethylase pathway and inhibited the accumulation of N-nitrosodimethylamine and its precursors in fermented fish products, and the final content was significantly lower than that in the spontaneous fermentation samples.

The storage method of preserved meat products in China mostly relies on room temperature shelves, so it is easy to observe stickiness, odor, acid production, and other corruption and deterioration during storage, which seriously affects the storage circulation and quality safety of the products. In recent years, the research on prolonging the storage time of preserved meat products has mainly focused on temperature control [29], packaging methods [30], or adding extracts [31,32]. Microbial fermenters, as a common control means, can control the growth of foodborne pathogens and spoilage bacteria in cured meat products [33,34], delay the occurrence of contamination when the phenomenon of contamination starts to occur in meat products [35], and prolong the shelf life of food products. In this paper, we produced three groups of sauced meat, including the no-addition group (CK), the *S. cerevisiae* group (SC), and the *K. marxianus* group (KM), and compared the physicochemical, sensory characteristics, and safety of these products during storage. The application of microbial fermentation technology is expected to provide the theoretical and practical basis for the practical problems in the storage and circulation of sauce meat.

## 2. Materials and Methods

### 2.1. Materials and Reagents

Experiment materials: pork belly, Sichuan Gaojin Industrial Group Co., Ltd. (Suining, China); S. cerevisiae, K. marxianus, Angie’s Yeast Ltd. (Yichang, China).

Reagents: potassium hydrogen phthalate (pH = 6.86 buffer), Aoran Institute of Fine Chemical Industry (Tianjin, China); mixed phosphate (pH = 4 buffer), Aoran Institute of Fine Chemical Industry (Tianjin, China); potassium chloride, sodium hydroxide, trichloroacetic acid, zinc acetate, disodium ethylene diamine tetraacetic acid (disodi-um EDTA), thiobarbituric acid (TBA), petroleum ether, sodium thiosulfate, and other reagents were purchased from Sichuan Kelon Chemical Co., Ltd. (Chengdu, China) Coomas G-250 stain, Ninhydrin stain, Yuanye Biotechnology Co., Ltd. (Shanghai, China) Ethyl maltol, Weisheng Long Food Co., Ltd. (Beijing, China). 

### 2.2. Instruments and Equipment

Tumbler (BVBJ-60L), Jiaxing Aibo Industry Co., Ltd. (Hangzhou, China) Vacuum Packaging Machine (GY-ZB-6202), Jiangxi Gan Yun Food Machinery Company (Ganzhou, China); Moisture Activity Measuring Instrument (HD-5), Huake Instrumentation Co. (ZFD-A5140) Wuxi, China; Zhicheng Analytical Instrument Co., Ltd. (Shanghai, China); Testo 205 pH meter, German Instrument Co., Ltd. (Shenzhen, China); Ultraviolet spectrophotometer (UV-1100), Mepheda Instrument Co. (HHS-11-4), Shanghai, China; Boxun Industrial Co., Ltd. (Shanghai, China).

### 2.3. Sample Preparation

Raw material: 1000 g of pork, 50 g of spices (sweet flour paste, cinnamon, cloves, star anise, cumin, Sichuan pepper), 20 g of white wine, 0.4 g of ethyl maltol, 0.05 g of sodium nitrite.

**Figure 1:**
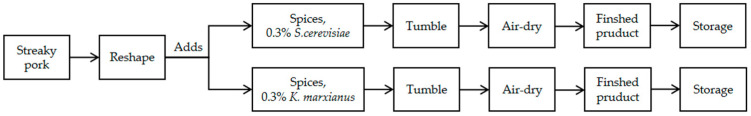
Flowchart of the process of making sauced meat.

Production process: Follow the process of making sauced meat in Figure 1. Streaky pork was chosen, cut into uniform-sized strips (about 5 cm × 4 cm × 2 cm), and separated into three groups. A control group without added yeast (CK), a group with *S. cerevisiae* added at 0.3% of the mass of the raw meat (SC), and a group with *K. marxianus* added at 0.3% of the mass of the raw meat (KM) were created. The pork strips were placed into the vacuum tumbler after being spiced, tumbled for 30 min at 4 °C (tumbling for 5 min and standing for 10 min), and then stood under anaerobic conditions for 12 h at 25 °C. After shaping, the meat strips were placed in an air-drying and fermentation apparatus and hung on a rope while the temperature, air velocity, and humidity were regulated to 10.0 °C, 1.0 m/s, and 65%, respectively. Air-drying was then carried out for 8 days. 

The prepared sauce meat was stored at room temperature at 20 °C for six months after vacuum packing in vacuum bags, and samples were collected at months 1, 2, 4, and 6 for repeated measures analysis.

### 2.4. Determination of Moisture Content and Water Activity

After mincing, the sauced meat with a balanced ratio of fat to lean mass, is accurately weighed to 3 g in the sample tray of the moisture tester to determine the moisture content. Then, the average of the results of three parallel measurements is recorded. In order to determine moisture activity using the GB 5009.238-2016 “National Food Safety Standards for the Determination of Food Moisture Activity” [36] method, a sample of approximately 2 g must be homogenized and spread out evenly over the moisture activity meter in a petri dish. Next, the average value from three separate parallel determinations is taken.

### 2.5. Determination of pH

The pH meter was used to make a direct measurement in accordance with GB/T 5009.237-2016, “Determination of pH value of foodstuffs of national food safety standards” [37].

### 2.6. Determination of Malondialdehyde, Acid Value, and Peroxide Value

Malondialdehyde was determined using the method of Liu [38]. After measuring the treated sample’s supernatant’s absorbance at 532 and 600 nm, the TBARS was calculated using the following formula:

TBARS(mg/100 g) = (*A*_532_ − *A*_600_)/155 × 0.1 × 72.6 × 100 (1)

In accordance with GB/T 5009.229-2016, “Determination of acid value in Foodstuffs, the National Standard for Food Safety” [39], the acid value was directly established. The sample (3 ± 0.0001) g was weighed into a conical bottle, 50 mL ether–isopropanol mixture (V:V = 1:1) was added, three drops of phenolphthalein indicator were added, and the sample was titrated with 0.1 mol/L KOH solution until the pink color does not disappear within 15 s as the end point of titration.

The peroxide value was determined by crushing the sample and adding 2~3 times the volume of petroleum ether, mixing thoroughly, and then leaving it to macerate for more than 12 h. The filtrate was filtered through a funnel containing anhydrous sodium sulfate, and the residue of petroleum ether was evaporated by a rotary evaporator as the sample to be tested. Finally, (3 ± 0.0001) g of the sample to be tested was weighed according to the titration method in GB/T 5009.227-2016 “Determination of Peroxide Value in Foods in the National Standard for Food Safety” [40].

### 2.7. Determination of Free Amino Acid Content

Determination by the colorimetric method of ninhydrin concerning Zhang et al. [1].

Scale curve equation:

y=0.0096x−0.0145

where *x* is amino acid content (μg/mL) and *y* is absorbance (nm).

Correlation coefficient:

$$R^{2}=0.9953$$

Free amino acid content:

(2) $$\;(mg/100\;g)=\frac{m1}{m2\times 10}$$

In Equation (2), *m*1 is the sample free amino acid content found by the standard curve, and *m*2 is the sample mass (g).

### 2.8. Determination of Soluble Protein Content

Referring to the method of Zhu et al. [41] the absorbance was measured at 595 nm after sample treatment.

Scale equation: y=0.0396x+0.0329

where *x* is protein content (μg/mL), *y* is absorbance (nm) 

Correlation coefficient: $R^{2}=0.9974$

(3) $$\mathrm{Soluble}\;\mathrm{protein}\;\mathrm{content}\;(\mu g/g)=\frac{n1}{n2}$$

In Equation (3), *n*1 is the soluble protein content of the sample found by the standard curve (g) and *n*2 is the sample mass (g). 

### 2.9. Determination of Volatile Flavor Substances

Pre-treatment conditions: Take 3 g of the pulverized sample and place it in a 20 mL headspace bottle. Add 1 μL of the 2 μg/L 2,4,6-trimethylpyridine standard solution. Set up the CTC autosampler for the pre-treatment of the sample under the following conditions: heating box temperature, 75 °C; heating time, 35 min; sample extraction time, 20 min; and resolution time, 5 min. Conditions for the GC include: an HP-5MS UI column (30 m × 0.25 mm, 0.25 μm), a pressure of 32.0 kPa, a flow rate of 1.0 mL/min, and a carrier gas He gas, non-shunt injection; inlet temperature, 250 °C; the temperature increase program: the temperature increased from 40 °C to 85 °C at a rate of 3 °C per minute, held for 1 min, and then grew to 150 °C at a rate of 3 °C per minute, kept for 2 min, and then increased to 230 °C at a rate of 20 °C per minute. MS parameters include an electron ionization source (EI), electron energy of 70 eV, ion source and quadrupole temperatures of 230 °C and 150 °C, respectively, detector voltage of 350 V, and a mass scan range (*m*/*z*) of 40~500. 

Characterization: The operation was completed using identification of volatile substances utilizing the automatic deconvolution system of the GC-MS coupler workstation specifically designed for the task. The compound data were searched for and compared against the NIST 14.

### 2.10. Data Processing

The data from each group of experiments repeated three times were statistically analyzed using Microsoft Excel 2021 to prepare the data, and one-way analysis of variance (ANOVA), multiple comparisons (LSD Duncan), and principal component analysis were performed using the SPSS26.0 software, with a significant difference of *p* < 0.05. GraphPad Prism 9.0 and SIMCA 14.1 (32-bit) software were used to plot the physicochemical and flavor changes of the sauce meat during storage.

## 3. Results and Discussion

### 3.1. Physical and Chemical Indicators

#### 3.1.1. Changes in Moisture Content and Water Activity

**Figure 2:**
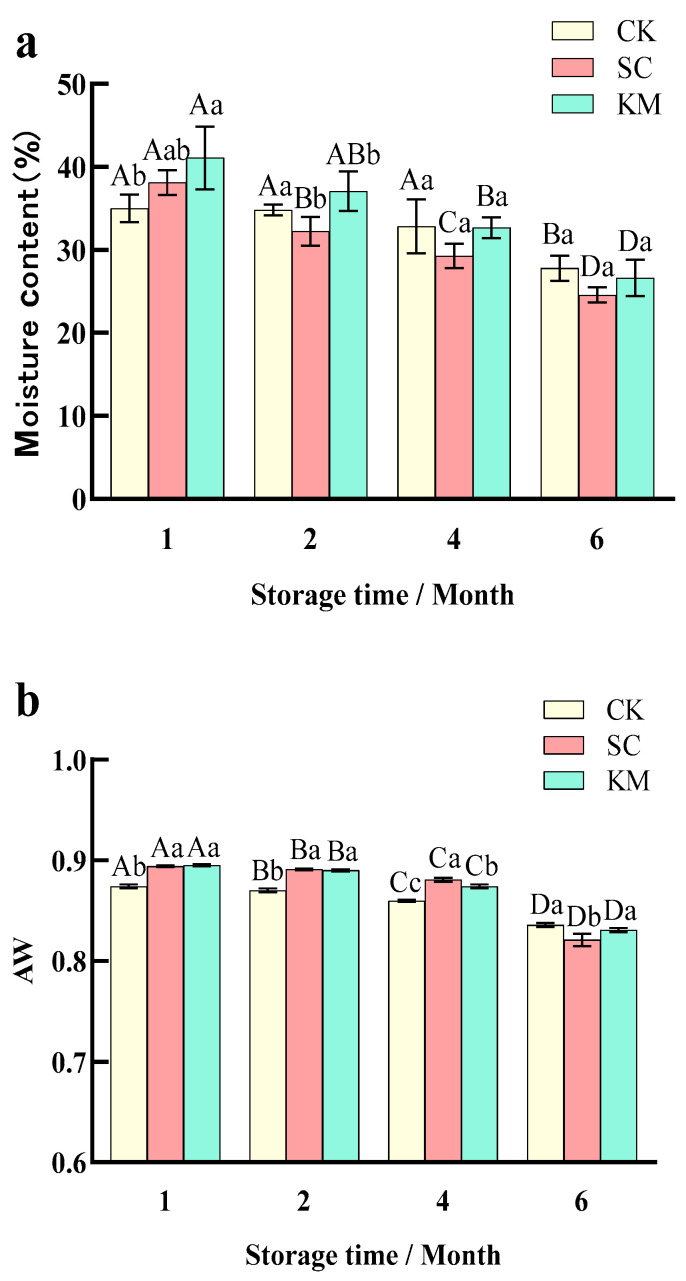
(**a**) Changes in moisture content in sauce meat during storage with the addition of different yeasts. (**b**) Changes in water activity in sauce meat during storage with the addition of different yeasts. A control group without added yeast (CK), a group with *S. cerevisiae* added at 0.3% of the mass of the raw meat (SC), and a group with *K. marxianus* added at 0.3% of the mass of the raw meat (KM). Note: capital letters represent the difference between the same groups at different times (*p* < 0.05), and small letters represent the difference between the groups at the same time (*p* < 0.05).

According to Figure 2, the moisture content and water activity in the sauce meat under the three treatments gradually decreased as storage time increased. This was in line with the findings of studies by Chai [42] and Wang [43], and it is most likely because of moisture loss brought on by high drying temperatures and low relative humidity during storage. The moisture level of the CK group dropped to 27.8% after 6 months of storage, whereas the moisture contents of the SC and KM groups that had added yeast fell to 24.54% and 26.63%, respectively. The initial moisture content was higher in the yeast-added group compared to the CK group in the 1st month of the storage period, but continued to decrease in the middle and late stages, reducing the variability (*p* > 0.05) with the other two groups, suggesting that brewer’s yeast may have a role in stabilizing the moisture content of the sauced meat. The drop in pH, which caused the muscle proteins in the sauce meat to gel and reduce its ability to retain water, was linked to the drop in aw [44,45,46]. The water activity of the SC and KM groups gradually decreased and was lower than that of the CK group in the later period; especially, the water activity of the SC and KM groups had a similar trend of change, which indicated that neither of the two types of yeasts added to the sauce meat negatively affected the quality of the sauce during the four months of storage (*p* > 0.05).

#### 3.1.2. Changes in pH Value

**Figure 3:**
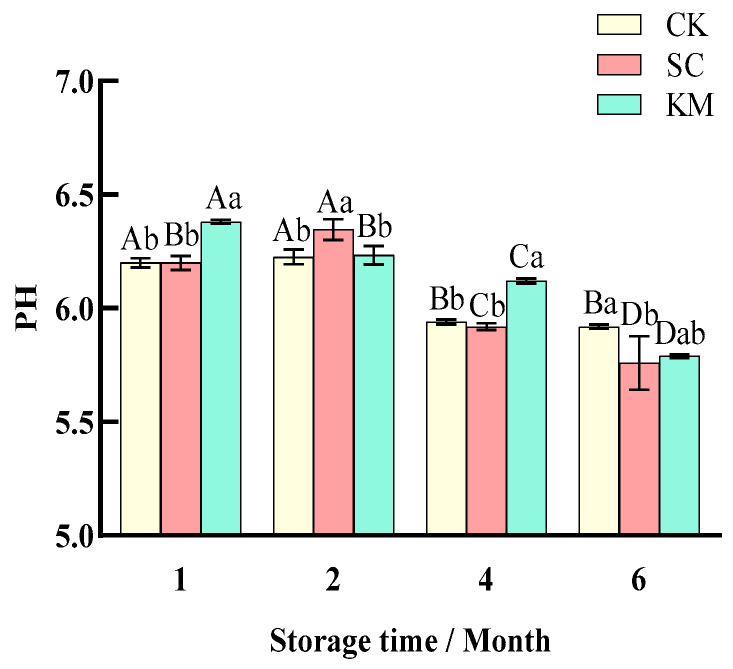
pH changes in sauce meat during storage with the addition of different yeasts. A control group without added yeast (CK), a group with *S. cerevisiae* added at 0.3% of the mass of the raw meat (SC), and a group with *K. marxianus* added at 0.3% of the mass of the raw meat (KM). Note: capital letters represent the difference between the same groups at different times (*p* < 0.05), and small letters represent the difference between the groups at the same time (*p* < 0.05).

The moisture content, water activity, and water holding capacity of the sauce meat [47,48], among other factors, directly influenced how the pH value of the sauce meat changed during storage. The pH values of the three groups of sauce meat in Figure 3 did not significantly change during the two months, and the drop in pH during the pre-storage period may have been brought on by the buildup of lactic acid brought on by the breakdown of carbohydrates during storage. The pH value of the CK group was higher than that of the control group at the time of storage by up to 6 M, and it decreased to 5.76 and 5.79 in the SC and KM groups, respectively. The synergistic action of nitrogen compounds and lactic acid bacteria under the two situations of increasing storage duration and hydrolysis of proteins for acid formation may be the cause of the general falling trend in the pH of the samples with increasing storage time [49,50].

The pH readings of each group had distinct declining trajectories, as shown in Figure 3, and at the end of storage, the pH of the sauced meat inoculated with *S. cerevisiae* was significantly lower than that of the *K. marxianus* group (*p* < 0.05). *S. cerevisiae* was the most efficient at reducing the pH of the sauce meat and making it easier to store because it prevented the formation of spoiling bacteria in the sauce meat. Low pH may nevertheless prevent yeast development and have an impact on the flavor of the sauce meat even though yeast has a high acid tolerance and can grow and live at acidic pH [51,52].

#### 3.1.3. Changes in AV, TBARS, and POV Values

**Figure 4:**
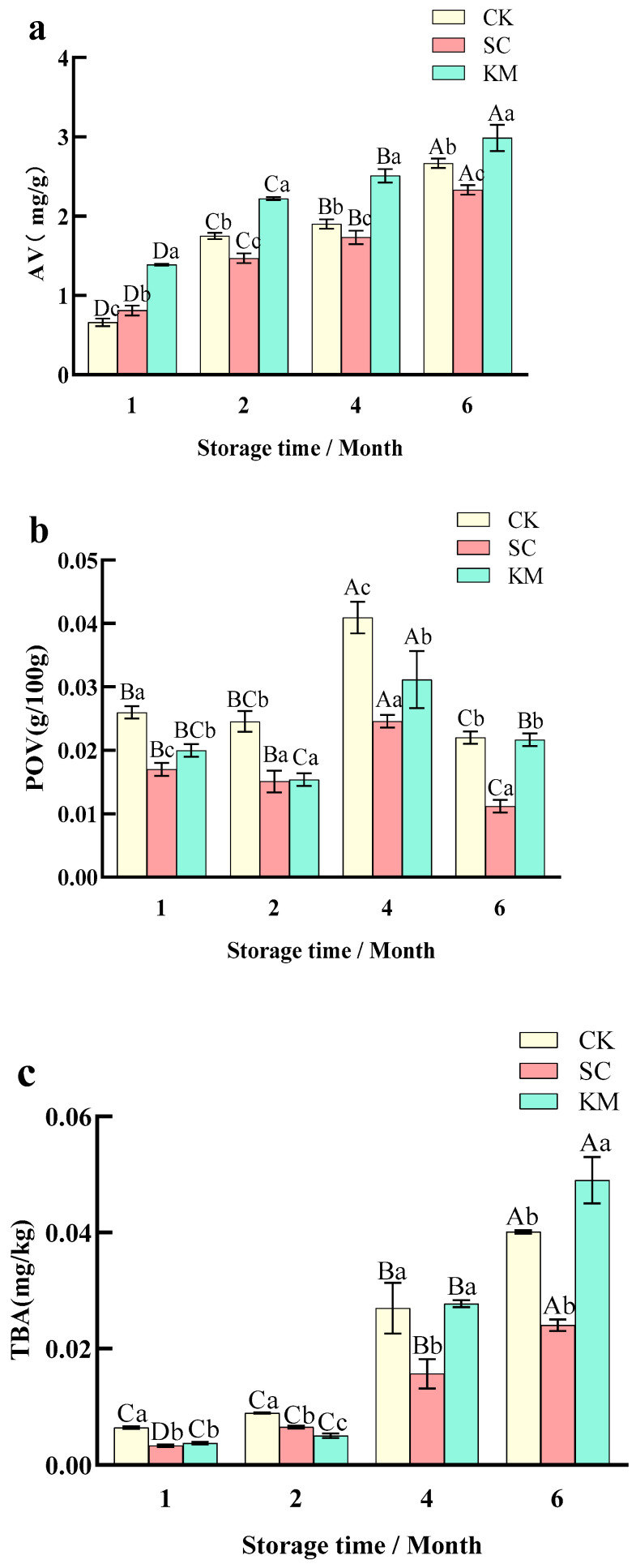
(**a**) Changes in TBARS values of sauce meat during storage with the addition of different yeasts. (**b**) Changes in acid price in sauce meat during storage with the addition of different yeasts. (**c**) Changes in peroxide value in sauce meat during storage with the addition of different yeasts. A control group without added yeast (CK), a group with *S. cerevisiae* added at 0.3% of the mass of the raw meat (SC), and a group with *K. marxianus* added at 0.3% of the mass of the raw meat (KM). Note: capital letters represent the difference between the same groups at different times (*p* < 0.05), and small letters represent the difference between the groups at the same time (*p* < 0.05).

The AV, TBARS, and POV values were all closely related to the fat quality of the sauce meat during storage, and a certain degree of lipid oxidation could help to accumulate the aromatic substances in the sauce meat, but excessive oxidation would lead to an off-flavor and deterioration of the sauce meat. As seen in Figure 4, during the whole storage period, the total content of the CK group fluctuated more, and the total content of the KM and SC groups fluctuated less. The AV and TBARS values of the sauce meat without added yeast were significantly higher than those of the sauce meat with added yeast, which was consistent with the changes in the moisture content of sauce meat in this experiment and the results of Zhang et al. [1]. It can be noted that the AV value of the SC group was lower than that of the other two groups and was more stable during the storage period (Figure 4a), which indicated that *S. cerevisiae* had a certain antioxidant effect during the storage process of sauce meat, and it could also prevent the excessive oxidation of food products from producing an undesirable odor [48,53]. 

In Figure 4c, the TBARS values formed during the storage of sauce meat from all three treatments were in the range of 0.23–0.70 mg/kg, and the larger the TBARS value, the more serious the fat oxidation. The TBARS values of the sauce meat in the CK, SC, and KM groups were approximately 0.0064, 0.0033, and 0.0037 mg/kg (*p* < 0.05) at 1M, respectively, and the three groups’ TBARS values abruptly increased to 0.027, 0.016, and 0.027 mg/kg (*p* < 0.05) at 4M, respectively, which may have been caused by changes in the sauce meat’s internal moisture. Changes in quality instability and secondary metabolites from fat oxidation were caused by internal dampness. The POV values of the sauce meat gradually increased over time, with the greatest fluctuation occurring between 2M and 4M, and peaking at 4M [54]. Though the rate of decomposition was faster than the rate of creation, with a drop in the POV value [55], as storage time accumulated, the breakdown of peroxides increased and degraded into tiny molecules, such as aldehydes and ketones [56].

#### 3.1.4. Changes in FAA Values

**Figure 5:**
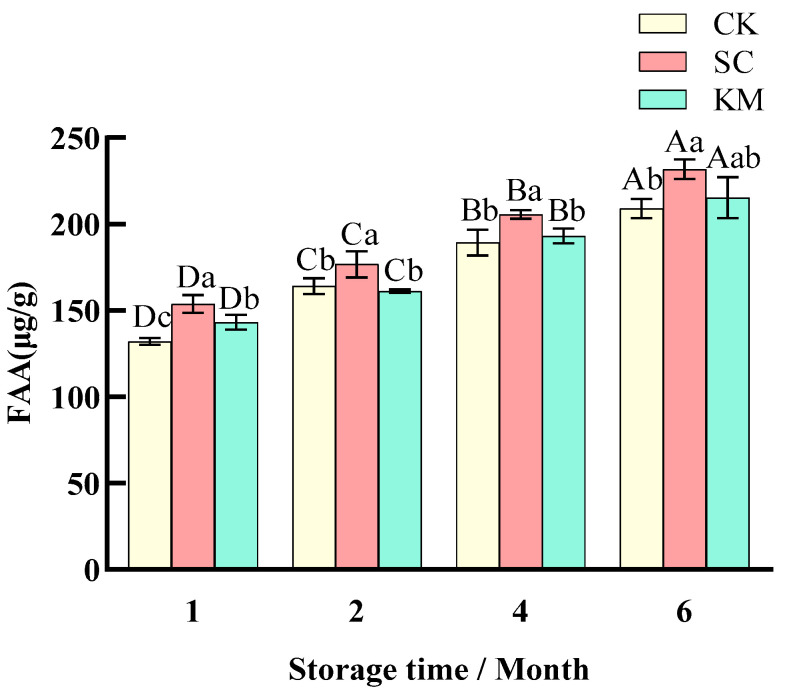
Changes in free amino acid content in sauce meat during storage with the addition of different yeasts. A control group without added yeast (CK), a group with *S. cerevisiae* added at 0.3% of the mass of the raw meat (SC), and a group with *K. marxianus* added at 0.3% of the mass of the raw meat (KM). Note: capital letters represent the difference between the same groups at different times (*p* < 0.05), and small letters represent the difference between the groups at the same time (*p* < 0.05).

Figure 5 shows that the FAA of sauce meat increased significantly (*p* < 0.05) over the course of six months, with the FAA of the SC group increasing from 153.73 μg/g at the first month of storage to 231.76 μg/g at six months. There was no significant difference in the FAA content of the CK and KM groups, which were each 209 and 215.24 μg/g, respectively, at the time of storage up until June. For the purpose of creating sauce meat and expanding sauce meat with storage time, air-drying, fermentation, and spice addition treatments were used. The biggest change in FAA content between the 1st and 2nd month during the storage period could be attributed to the more active yeast growth at the early stage of storage, and the increase in FAA with storage time was primarily caused by proteolysis, which is the process of protein breakdown through the action of endogenous enzymes, such as histone protease and calpain. Protein actions result in the breakdown of larger molecules into smaller ones (peptides, FAAs, and aldehydes) [57]. FAA is the main compound produced by protein hydrolysis, which is also the basis for the development of flavor in sauce meat. The greatest FAA level in the SC group in the figure suggests that the addition of *S. cerevisiae* increased the amount of protein hydrolysis in the sauce meat while also allowing the yeast to access free amino acids from the peptides and proteins in the sauce meat [11]. The sauce meat’s flavor was enhanced by the mixture of VOCs and FAAs formed during preservation using air-drying and fermentation methods.

#### 3.1.5. Changes in SP Values

**Figure 6:**
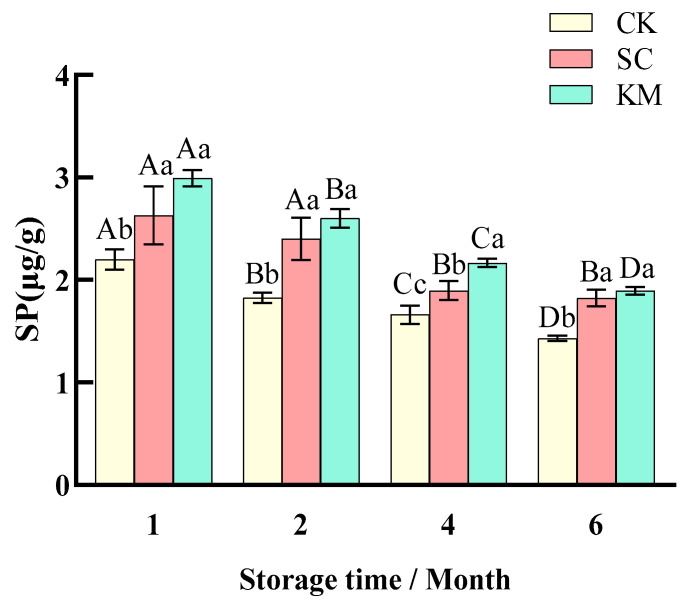
Changes in soluble protein content in sauce meat during storage with the addition of different yeasts. A control group without added yeast (CK), a group with *S. cerevisiae* added at 0.3% of the mass of the raw meat (SC), and a group with *K. marxianus* added at 0.3% of the mass of the raw meat (KM). Note: capital letters represent the difference between the same groups at different times (*p* < 0.05), and small letters represent the difference between the groups at the same time (*p* < 0.05).

Moderate protein breakdown to peptides, amino acids, aldehydes, and other low molecular mass components during the fermentation and preservation of meat products may significantly enhance the flavor and nutritional value of sauce meat [58,59]. However, excessive protein oxidation may negatively impact the texture, color, and flavor of sauce meat. Protein oxidation is a free radical chain reaction similar to lipid oxidation [60], but the targets and pathways of protein oxidation are more complex and closely related to the type and nature of the oxidation products [61]. The exogenous proteases produced by *K. marxianus* were mostly responsible for the variations in SP levels in this experiment. The SP value of the sauce meat after yeast addition was significantly higher (*p* < 0.05) than that of the control group (Figure 6), and the content was decreased from 2.2 μg/g to 1.43 μg/g in the CK group, and from 2.63 μg/g and 2.99 μg/g to 1.82 μg/g and 1.89 μg/g in the SC and KM groups, respectively. In some cases, the structural alterations brought on by the slow oxidation of proteins during storage might lower the sauce meat’s product value and even cause it to deteriorate and spoil, which poses a severe threat to its suitability for human consumption.

### 3.2. Flavor Change Rule

As shown in Table 1, a total of 111 volatile flavor substance components were identified in the storage process of sauce meat, of which 23, 26, 20, and 33 substances were detected during the first, second, fourth, and sixth months in the CK group, respectively; 32, 44, 29, and 49 substances were detected during the first, second, fourth, and sixth months in the SC group, respectively; and 37, 39, 40, and 41 substances during the first, second, fourth, and sixth months in the KM group, respectively. Among the various flavor substances, the substances in the categories were alcohols, esters, and olefins, with 17, 20, and 33 substances, accounting for 15.32, 18.01, and 29.73% of the total number of species, respectively, followed by aldehydes (12), acids (6), and alkanes (11), accounting for 10.81, 5.4, and 9.9% of the total number of species, respectively. The results of this study were similar to those of the studies conducted by Zhou [62] and Wang et al. [63]. 

**Figure 7:**
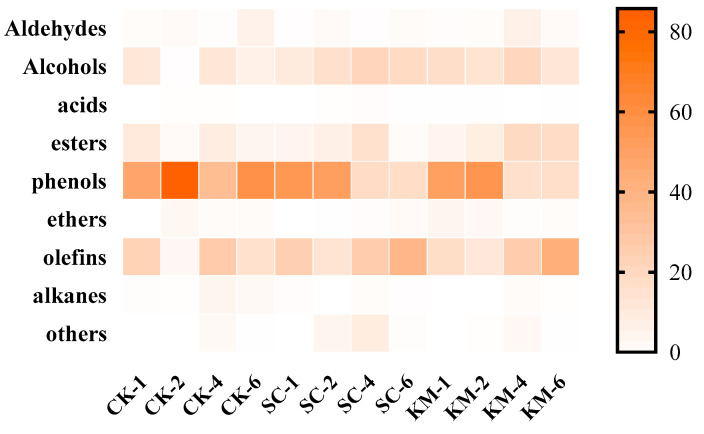
Heat map of various volatile flavor types.

During the making and preservation of sauce meat, lipid oxidation, amino acid catabolism, penetration of low-molecular-weight chemicals, esterification, and the Meladic reaction take place to create a distinctive flavor and release a range of volatile substances [64]. According to headspace solid-phase microextraction (SPME)–gas chromatography–mass spectrometry (SPME–GC–MS) analysis of the sauce meat in the four treatment groups, the amount of volatile flavoring compounds increased with storage time. Figure 7 demonstrates that phenols, alcohols, and olefins were the compounds with the largest quantity in sauce meat. The pattern of change in the flavor compounds in the sauce meat after storage was typically a rise in aldehydes and olefins, and a progressive reduction in esters and phenols. It is noteworthy that during the 6-month storage period, the phenolic content was constant and the alkenes decreased in the CK group; the alcohols were elevated significantly in the SC group; and the esters in the KM group had the highest relative content at the end of storage, which is similar to the results of the study on *K. marxianus* by Gao [26] and Li [65].

In this investigation, the only two types of phenolic chemicals found in the sauce meat were ethyl maltol and eugenol. As a safe and reliable food additive with a caramel aroma and fruity flavor, ethyl maltol increased the sweet aroma of the sauce meat to form a distinctive flavor that is different from that of bacon. Ning et al. [66] investigated the compound seasoning with a high starting acid value as a research object and found that ethyl maltol also affects the flavor of the compound seasoning. The acid value increases with the amount of ethyl maltol added. The AV value of the sauce meat displayed comparable findings that may have been impacted by the fact that the ethyl maltol level of the CK and KM groups was much greater than that of the SC group, as shown in Table 2. When compared to the CK and KM groups, the second-largest group of substances—alcohols—increased rather than reduced in the infected *S. cerevisiae* sauce meat, and linalool, which was the most prevalent alcoholic compound in the SC group, rose by 44.60%. The inclusion of linalool during the production process can lower the contamination of *Salmonella* spp., *Listeria monocytogenes*, and *Escherichia coli* [67]. Linalool has a light and fresh floral and woody scent. As unsaturated fatty acids break down over a longer period of time while being stored, alcohol levels rise, giving the sauce meat a distinctive flavor. In the study of alcohols, Li [68] on Longxi bacon also included 1-octen-3-ol and linalool as the primary volatile flavoring compounds. *S. cerevisiae* encourages the production of esters, particularly medium-chain fatty acid esters (MCFA) and ethyl esters [69,70], Lv [71]. The VOCs content of the group with the addition of *S. cerevisiae* at the end of the fermentation was 4.36-fold higher than that of the blank group (*p* < 0.01), which is significantly higher than that of the blank group, according to the results of experiments with the addition of *S. cerevisiae* LXPSC1 to sour meat. Li [65] discovered that *K. marxianus* increases the quantity of flavoring compounds in yogurt and encourages the metabolism of lipids in yogurt. The highest concentration of esters in sauce meat in this experiment was linalyl acetate, which has a nice flowery and fruity aroma. It made up approximately 87.94% and 92.22% of the total ester content in the SC group (6M) and KM group (6M), respectively. As a result, it is assumed that *K. marxianus* and *S. cerevisiae* were added to increase the synthesis of esters.

Table 2 shows that the types of volatile chemicals in the beef with added yeast sauce were very different from those in the control group. Citral, -(+)-citronellal, styrene, camphene, hinokiene, dodecanal, and phenylethanol were the only ones of these substances that were only found in the experimental group. The most volatile flavor compounds were found in the SC group at storage until 6M, including nonanoic acid, ethyl isovalerate, citral, camphene, hinokiene, and hexanal, which significantly improved the flavor of the sauce meat. Linalool, -pinitol, linalyl acetate, and lauroylenol were significantly higher in the KM group than in the CK group.

**Figure 8:**
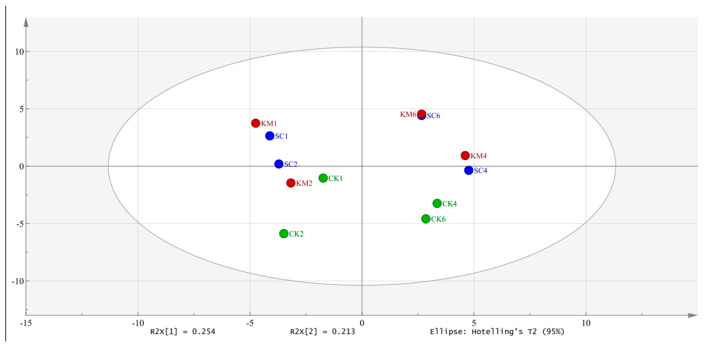
PCA plots of key flavor substances in sauce meat (A). A control group without added yeast (CK), a group with *S. cerevisiae* added at 0.3% of the mass of the raw meat (SC), and a group with *K. marxianus* added at 0.3% of the mass of the raw meat (KM).

**Figure 9:**
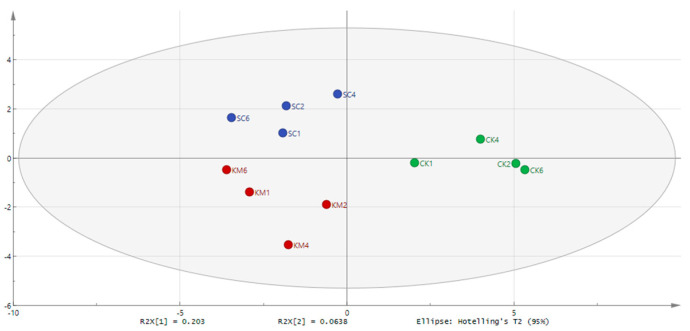
PLS-DA model for key flavor substances in sauce meat (B). A control group without added yeast (CK), a group with *S. cerevisiae* added at 0.3% of the mass of the raw meat (SC), and a group with *K. marxianus* added at 0.3% of the mass of the raw meat (KM).

The variations in volatiles between blank meat and sauce meat that had been inoculated with yeast were reflected in the PCA spatial distribution maps. In the PCA maps (Figure 8), there were notable differences between the SC and KM groups of sauce meat that had been held until the fourth and sixth months, and the blank sauce meat. There was also some overlap between the KM6 and SC6 PCA maps. Screening volatile flavor molecules that are crucial to the process of flavor recognition can be completed based on the PLS-DA. Figure 9 shows the results of the three sets of samples’ GC-MS analyses were analyzed through Simca’s partial least squares discriminant analysis (PLS-DA) for the associated flavor chemicals. The 24 volatile compounds included fennel brain, camphene, hinokiene, P-umbelliferyl hydrocarbons, heptanal, -rhododendron, n-hexanal, A-limonene, nonanoic acid, -erythromycene, turpentine acetate, geranyl acetate, linalyl acetate, -stigmasterol, and -Aniseed brain, which plays a significant role in the flavor development of sauce meat. During curing and storage, spices like fennel and star anise produce flavor compounds that are transferred to the sauce meat [72]. Other distinctive aromas of star anise, such as geranyl acetate, geranyl acetate, linalyl acetate, and -geranylene, are also distinctive flavor compounds in the sauce meat. In general, the esters, aldehydes, and hydrocarbons below mostly represent the changes in volatility between sauce meat inoculated with and without yeast. Some aldehydes may be converted to acids during the first phases of sauce meat’s natural fermentation, and these acids then combine with alcohols to create esters [73]. Citral has a sweet, warm, pungent lemony flavor, whereas -(+)-citronella aldehyde has a powerful, fresh, and somewhat woodsy flavor with a touch of sweetness less than citral. Aldehydes have a strong odor and a low threshold, and they are also a significant component of the meat flavor. Linalool and linalyl acetate both have flavors that are sweet, fresh flowery, and citric [74]. Linalool can be produced using *S. cerevisiae*, and when the sauce meat was stored for a longer period of time, the concentrations of linalool and linalyl acetate were much greater in the SC and KM groups than in the CK group. Using the heterologous expression of linalool synthase, IUP, and the ERG20 mutation in the yeast *S. cerevisiae*, Zhang et al. [75] effectively created *S. cerevisiae*. To improve the synthesis of linalool from *S. cerevisiae*, yeast has been used as a platform. *S. cerevisiae* and *K. marxianus* added to sauce meat improved the quality and flavor stability over long-term storage.

## 4. Conclusions

By using GC-MS to compare the effects of adding *S. cerevisiae* and *K. marxianus* to sauce meat, the differences between the two experimental groups were clearly discernible. The data revealed a significant increase in alcohols, esters, and olefins with the addition of the yeasts, indicating that the two types of yeasts significantly enhanced the flavor components of the sauce meat during storage (*p* < 0.05). The experimental group (SC group) significantly decreased the pH, AV, and TBARS values of the sauce meat (*p* < 0.05), and the POV and TBARS values of the SC group decreased by 49.09% and 40.15%, respectively, compared to the CK group at the time of storage up to six months, indicating that *S. cerevisiae* could reduce the degree of lipid oxidation in the storage process of the sauce meat, and it has the potential to add to the flavor of the sauce meat. The experimental group (KM group) significantly increased the SP and FAA values of the sauce meat (*p* < 0.05) by 32.4% and 29.84%, respectively, compared with the CK group, indicating that the microbial proteases in *K. marxianus* can promote the proteolysis of sauce meat and increase the soluble protein and free amino acid content of the sauce meat.

However, separately, the two kinds of yeast have their own merits in sauce meat, so the two yeasts can be mixed or added with other fermentation agents in sauce meat in the extension project. At the same time, we can explore in-depth the specific mechanism of the yeast to improve the quality and safety of the sauce meat. We hope to provide high-quality fermentation microorganism resources for the factory production of sauce meat.

## Figures and Tables

**Table 1:** Types and contents of volatile flavor substances in various types of sauced meat.

Sample	Time (Month)	Compounds	Aldehydes	Alcohols	Acids	Esters	Phenols	Ethers	Olefins	Alkanes	Others
CK	1	relative content (%)	1.4	12.87	0	11.53	49.04	0	24.22	0.94	0
		amount	3	3	0	4	2	0	10	1	0
	2	relative content (%)	2.46	0.56	0.43	2.18	85.72	4.24	3.87	0.53	0
		amount	5	2	2	4	2	1	7	3	0
	4	relative content (%)	0.84	13.17	0.49	9.77	37.54	2.12	28.2	5.18	2.7
		amount	1	2	1	5	1	1	3	6	0
	6	relative content (%)	6.62	7.54	0	5.24	59.42	1.97	16.23	2.76	0.23
		amount	5	5	0	7	1	1	7	6	1
SC	1	relative content (%)	0.57	11.07	0	5.03	56.23	0	25.92	1.19	0
		amount	2	6	0	4	2	0	17	1	0
	2	relative content (%)	2.31	16.97	0.43	7.74	52.36	0.13	14.77	0	5.3
		amount	8	7	3	6	2	0	15	0	3
	4	relative content (%)	0.66	22.85	1.18	16.41	19.18	1.13	27.08	1.37	10.14
		amount	1	6	1	7	1	1	8	4	0
	6	relative content (%)	1.69	19.86	0.15	17	18.56	2.28	39.13	0.61	0.72
		amount	5	9	2	9	2	1	15	4	2
KM	1	relative content (%)	1.08	18.05	0.12	5.53	52.09	4.86	18.27	0	0
		amount	4	9	1	3	2	1	17	0	0
	2	relative content (%)	1.38	15.08	0.24	9.13	57.37	3.6	12.69	0	0.51
		amount	5	7	1	7	2	2	12	0	3
	4	relative content (%)	7.68	21.64	0	20.11	16.81	0.96	27.23	1.92	3.65
		amount	3	7	0	9	1	1	11	4	4
	6	relative content (%)	2.39	13.71	0.06	18.98	18.87	1.61	43.84	0.42	0.12
		amount	4	6	1	5	2	1	16	4	2

**Table 2:** Statistics on the relative content of volatile compound components in the sauce meat during the storage period.

RT	Name	Relative Content (%)											
		CK				SC				KM			
		1	2	4	6	1	2	4	6	1	2	4	6
	Aldehydes (12)	1.40	2.46	0.84	6.62	0.57	2.31	0.66	1.69	1.08	1.38	7.68	2.39
4.38	Hexanal	-	-	-	1.62	-	-	-	0.44	-	-	6.03	0.71
7.34	Heptanal	-	-	-	0.34	-	-	-	-	-	-	0.56	0.15
7.74	Methional	0.06	0.25	-	0.16	-	0.06	-	-	-	-	-	-
9.76	Benzaldehyde	0.86	0.72	-	-	-	0.55	-	-	0.35	-	-	-
13.23	Benzeneacetaldehyde	0.48	1.02	-	2.40	0.38	0.52	-	0.11	0.17	0.50	-	0.10
15.94	Nonanal	-	0.26	0.84	2.10	-	0.34	0.66	1.06	-	-	1.09	1.37
15.95	10-Undecenal	-	-	-	-	-	-	-	-	-	0.31	-	-
15.99	Dodecanal	-	-	-	-	-	-	-	-	0.40	-	-	-
18.48	6-Octenal, 3,7-dimethyl-, (R)-	-	-	-	-	-	0.04	-	0.02	-	-	-	-
23.63	2, 6-Octadienal, 3,7-dimethyl-, (Z)-	-	-	-	-	-	0.25	-	-	-	0.18	-	-
25.13	2-Propenal, 3-phenyl-	-	0.20	-	-	-	0.15	-	-	-	0.12	-	-
25.27	Citral	-	-	-	-	0.19	0.40	-	0.06	0.15	0.28	-	0.05
	Alcohols (17)	12.87	0.56	13.17	7.54	11.07	16.97	22.85	19.86	18.05	15.08	21.64	13.71
3.24	1-Butanol, 2-methyl-	-	-	-	-	-	-	-	0.68	-	-	-	-
4.18	2,3-Butanediol, [R-(R*, R*)]-	-	-	-	0.37	-	-	-	-	-	-	-	-
6.34	1-Hexanol	-	-	-	-	-	-	-	0.22	-	-	-	-
12.48	Eucalyptol	-	-	3.77	1.63	1.11	1.89	3.51	4.34	0.89	1.48	2.88	2.51
14.13	Bicyclo[3.1.0]hexan-2-ol, 2-methyl-5-(1-methylethyl)-, (1.alpha., 2.beta., 5.alpha.)-	-	-	-	-	-	-	1.49	-	0.74	-	1.36	-
14.18	5-Isopropyl-2-methylbicyclo[3.1.0]hexan-2-ol	-	-	-	-	-	0.66	-	0.67	-	1.58	-	-
14.24	Bicyclo[3.1.0]hexan-2-ol, 2-methyl-5-(1-methylethyl)-, (1.alpha., 2.alpha., 5.alpha.)-	-	-	-	-	0.14	0.69	-	0.64	0.64	-	-	0.59
14.43	trans-Linalool oxide (furanoid)	-	-	-	-	-	-	-	0.25	0.12	-	-	0.13
14.47	.alpha.-Methyl-.alpha.-[4-methyl-3-pentenyl]oxiranemethanol	-	-	-	-	-	0.10	-	-	-	0.09	0.19	-
15.55	5-Isopropyl-2-methylbicyclo[3.1.0]hexan-2-ol	-	-	-	-	-	-	0.85	-	-	-	0.75	-
15.81	Linalool	10.78	-	9.41	4.55	7.35	10.49	13.66	10.62	12.27	9.17	13.28	8.05
16.41	Phenylethyl Alcohol	-	-	-	-	-	-	-	0.26	0.28	-	-	-
19.79	3-Cyclohexen-1-ol, 4-methyl-1-(1-methylethyl)-, (R)-	-	0.44	-	0.83	-	2.46	2.93	2.18	-	2.15	2.84	2.06
19.83	Terpinen-4-ol	1.57	-	-	-	1.75	-	-	-	2.40	-	-	-
20.65	.alpha.-Terpineol	0.51	0.12	-	0.16	0.66	0.68	0.42	-	0.63	0.54	0.34	0.37
30.43	Nerolidol	-	-	-	-	0.08	-	-	-	0.09	-	-	-
32.76	(1S,2R,5R)-2-Methyl-5-((R)-6-methylhept-5-en-2-yl)bicyclo[3.1.0]hexan-2-ol	-	-	-	-	-	-	-	-	-	0.07	-	-
	Acids(6)	0.00	0.43	0.49	0.00	0.00	0.43	1.18	0.15	0.12	0.24	0.00	0.06
6.11	Butanoic acid, 3-methyl-	-	-	0.49	-	-	-	-	-	-	-	-	-
6.56	Hexanoic acid, 2-methyl-	-	-	-	-	-	-	1.18	-	-	-	-	-
6.94	Formic acid	-	-	-	-	-	-	-	-	0.12	-	-	-
11.15	Hexanoic acid	-	-	-	-	-	0.16	-	-	-	-	-	-
25.72	Nonanoic acid	-	0.17	-	-	-	0.09	-	0.12	-	-	-	-
30.09	n-Decanoic acid	-	0.26	-	-	-	0.17	-	0.03	-	0.24	-	0.06
	esters(20)	11.53	2.18	9.77	5.24	5.03	7.74	16.41	17.00	5.53	9.13	20.11	18.98
3.61	Propanoic acid, 2-methyl-, ethyl ester	-	-	-	-	-	-	0.45	-	-	-	-	-
3.71	Hydrazinecarboxylic acid, phenylmethyl ester	-	-	-	-	-	-	1.44	-	-	-	-	-
4.45	Butanoic acid, ethyl ester	-	-	-	1.03	-	-	-	0.44	-	-	-	-
4.79	Propanoic acid, 2-hydroxy-, ethyl ester	-	-	-	0.41	-	-	-	0.23	-	-	-	-
5.73	Butanoic acid, 2-methyl-, ethyl ester	-	-	1.14	0.43	-	-	1.74	0.08	-	-	0.20	-
5.83	Butanoic acid, 3-methyl-, ethyl ester	-	-	3.06	0.68	-	-	3.51	0.11	-	-	0.26	-
7.39	Pentanoic acid, ethyl ester	-	-	0.53	-	-	-	-	-	-	-	-	-
9.42	Octyl chloroformate	-	-	-	-	-	-	-	-	-	-	0.18	-
9.59	2,5-Pyrrolidinedione, 1-(benzoyloxy)-	-	-	-	-	-	-	-	-	-	0.19	0.33	-
11.29	Hexanoic acid, ethyl ester	-	-	-	1.75	-	-	-	-	-	-	-	-
11.95	4-Terpinenyl acetate	-	-	-	-	0.23	-	-	-	-	-	-	-
24.54	Linalyl acetate	10.93	0.59	4.34	0.36	4.29	6.08	8.84	14.95	5.05	7.26	18.07	17.50
29.22	.alpha.-Terpinyl acetate	0.21	0.08	-	-	0.31	0.23	-	0.25	0.32	0.23	0.27	0.37
29.81	(R)-lavandulyl acetate	-	-	-	-	-	0.06	-	-	-	0.07	-	-
29.82	1,6-Octadien-3-ol, 3,7-dimethyl-, formate	0.14	-	-	-	-	-	-	-	-	-	-	-
30.33	2,6-Octadien-1-ol, 3,7-dimethyl-, acetate, (Z)-	-	-	-	-	-	-	0.12	0.14	-	-	0.13	0.19
30.35	Geranyl acetate	-	-	-	-	-	0.10	-	0.30	-	0.11	0.25	0.39
30.36	4-Hexen-1-ol, 5-methyl-2-(1-methylethenyl)-, acetate	0.25	-	-	-	0.20	-	-	-	0.16	-	-	-
30.46	Ethyl trans-4-decenoate	-	0.11	-	-	-	0.10	-	-	-	0.10	-	-
30.66	Decanoic acid, ethyl ester	-	1.40	0.71	0.59	-	1.16	0.30	0.50	-	1.18	0.42	0.53
	phenols(2)	49.04	85.72	37.54	56.23	52.36	52.28	19.18	18.56	52.09	57.37	16.81	18.87
21.49	Ethyl maltol	48.95	85.72	37.54	56.23	52.36	52.03	19.18	18.45	51.85	57.13	16.81	18.74
29.51	Eugenol	0.09	0.00	-	-	0.26	0.25	-	0.11	0.24	0.24	-	0.12
	ethers(2)	0.00	4.24	2.12	1.97	0.00	0.13	1.13	2.28	4.86	3.60	0.96	1.61
21.14	Estragole	-	-	-	-	-	0.13	-	-	-	0.10	-	-
25.94	Anethole	-	4.24	2.12	1.97	-	-	1.13	2.28	4.86	3.50	0.96	1.61
	olefins(33)	24.22	3.87	28.20	16.23	25.92	14.77	27.08	39.13	18.27	12.69	27.23	43.84
6.92	Styrene	-	-	-	-	-	-	-	-	-	-	0.09	-
9.10	Camphene	-	-	-	-	0.16	0.13	0.28	0.36	-	-	0.17	0.28
10.03	Bicyclo[3.1.0]hexane, 4-methylene-1-(1-methylethyl)-	-	-	-	-	-	0.15	-	0.78	0.25	0.14	1.08	1.71
10.86	.beta.-Pinene	-	0.73	-	-	-	-	-	-	-	-	-	-
10.96	.beta.-Myrcene	3.49	-	6.93	3.66	3.09	2.47	4.53	6.57	2.32	2.23	4.60	7.57
11.65	3-Carene	-	-	-	-	0.08	-	-	0.55	-	-	-	-
11.82	Cyclohexene, 1-methyl-4-(1-methylethylidene)-	-	-	-	-	-	-	-	-	-	-	-	1.31
11.95	1,3-Cyclohexadiene, 1-methyl-4-(1-methylethyl)-	0.48	0.11	1.04	0.89	-	0.54	1.01	-	0.44	0.26	1.39	-
12.23	Benzene, 1-methyl-3-(1-methylethyl)-	-	0.12	-	0.54	-	0.55	1.29	1.04	-	-	-	0.88
12.46	D-Limonene	14.41	2.58	20.23	9.16	13.10	8.11	17.13	24.55	10.40	7.68	16.49	27.40
12.99	(1R)-2,6,6-Trimethylbicyclo[3.1.1]hept-2-ene	0.22	0.11	-	0.80	0.56	0.79	-	1.96	0.41	0.65	0.20	1.04
13.36	1,3,6-Octatriene, 3,7-dimethyl-, (Z)-	-	0.10	-	0.40	-	-	0.39	-	-	-	0.65	-
13.45	.beta.-Ocimene	0.47	-	-	-	-	-	-	-	-	-	-	-
13.82	.gamma.-Terpinene	0.61	0.12	-	0.78	0.67	0.65	1.85	2.06	0.74	0.61	1.93	2.33
15.05	1,5,5-Trimethyl-6-methylene-cyclohexene	-	-	-	-	-	-	0.60	-	-	-	-	-
15.09	2-Carene	-	-	-	-	-	-	-	-	-	0.23	-	-
15.14	Cyclohexene, 1-methyl-4-(1-methylethylidene)-	0.32	-	-	-	-	-	-	-	0.42	-	-	-
17.13	2,4,6-Octatriene, 2,6-dimethyl-, (E,Z)-	0.26	-	-	-	0.17	-	-	-	-	-	-	-
25.91	Anethole	3.80	-	-	-	5.09	-	-	-	-	-	-	-
29.93	Copaene	-	-	-	-	0.14	0.05	-	-	0.07	-	-	0.07
30.76	Bicyclo[7.2.0]undec-4-ene, 4,11,11-trimethyl-8-methylene-	-	-	-	-	0.40	0.15	-	-	0.42	-	0.15	-
31.04	Caryophyllene	0.17	-	-	-	1.64	0.55	-	0.72	1.45	0.13	0.47	0.67
31.44	Bicyclo[3.1.1]hept-2-ene, 2, 6-dimethyl-6-(4-methyl-3-pentenyl)-	-	-	-	-	0.06	-	-	0.03	0.10	0.35	-	-
31.74	Humulene	-	-	-	-	-	-	-	0.04	0.13	-	-	0.05
32.34	Benzene, 1-(1, 5-dimethyl-4-hexenyl)-4-methyl-	-	-	-	-	0.31	0.33	-	0.14	0.44	0.25	-	0.16
32.49	1,3a-Ethano-3aH-indene, 1,2,3,6,7,7a-hexahydro-2,2,4,7a-tetramethyl-, [1R-(1.alpha., 3a.alpha., 7a.alpha.)]-	-	-	-	-	0.09	-	-	0.04	0.09	-	-	0.04
32.53	Di-epi-.alpha.-cedrene	-	-	-	-	-	-	-	-	-	-	-	0.14
32.55	1H-3a,7-Methanoazulene, 2,3,4,7,8,8a-hexahydro-3,6,8,8-tetramethyl-, [3R-(3.alpha., 3a.beta., 7.beta., 8a.alpha.)]-	-	-	-	-	-	-	-	-	0.23	0.09	-	-
32.55	Cedrene	-	-	-	-	0.14	0.12	-	-	-	-	-	-
32.76	.beta.-Bisabolene	-	-	-	-	0.09	0.09	-	0.21	0.14	-	-	0.11
32.97	1H-3a,7-Methanoazulene, octahydro-3,8,8-trimethyl-6-methylene-, [3R-(3.alpha., 3a.beta., 7.beta., 8a.alpha.)]-	-	-	-	-	-	-	-	0.08	-	-	-	0.09
32.99	Naphthalene, 1,2,3,5,6,8a-hexahydro-4, 7-dimethyl-1-(1-methylethyl)-, (1S-cis)-	-	-	-	-	0.12	0.10	-	-	0.21	-	-	-
32.99	.beta.-copaene	-	-	-	-	-	-	-	-	-	0.07	-	-
	alkanes(11)	0.94	0.53	5.18	2.76	1.19	0.00	1.37	0.61	0.00	0.00	1.92	0.42
10.62	Octane, 2,7-dimethyl-	-	-	-	-	-	-	-	-	-	-	1.54	-
10.65	Heptane, 2,2,4,6,6-pentamethyl-	-	-	2.91	-	-	-	0.89	-	-	-	-	-
26.73	Tridecane	-	0.25	-	0.37	-	-	-	-	-	-	-	-
29.87	Tridecane, 3-methyl-	-	0.14	-	-	-	-	-	-	-	-	-	-
30.67	Tetradecane	0.94	-	-	-	1.19	-	-	-	-	-	-	-
32.59	Pentadecane	-	0.15	0.49	0.23	-	-	0.22	-	-	-	0.17	-
33.99	Hexadecane	-	-	-	0.38	-	-	-	0.12	-	-	-	0.08
35.21	Heptadecane	-	-	0.29	0.68	-	-	-	0.18	-	-	-	0.12
36.31	Octadecane	-	-	0.55	0.70	-	-	0.13	0.21	-	-	0.10	0.15
37.34	Nonadecane	-	-	0.64	0.40	-	-	0.14	0.11	-	-	0.11	0.08
38.30	Eicosane	-	-	0.30	-	-	-	-	-	-	-	-	-
	others(8)	0.00	0.00	2.70	0.23	0.00	5.30	10.14	0.72	0.00	0.51	3.65	0.12
3.20	Oxirane, 2-(1,1-dimethylethyl)-3-methyl-	-	-	2.70	-	-	-	10.14	0.63	-	-	0.41	-
6.23	p-Xylene	-	-	-	-	-	-	-	-	-	-	0.23	0.04
3.71	Toluene	-	-	-	-	-	-	-	-	-	-	0.67	-
7.77	Furan, 2-ethyl-5-methyl-	-	-	-	0.23	-	5.12	-	-	-	-	-	-
12.23	o-Cymene	-	-	-	-	-	-	-	-	-	0.33	2.34	-
20.38	Naphthalene, 1,2,3,4,4a,5,8,8a-octahydro-4a-methyl-, trans-	-	-	-	-	-	0.13	-	-	-	0.13	-	-
29.87	2-Bromo dodecane	-	-	-	-	-	0.04	-	-	-	0.05	-	-
32.57	Hexadecane, 1-chloro-	-	-	-	-	-	-	-	0.09	-	-	-	0.07

Note: “-” indicates not detected.

## Data Availability

Data is contained within the article.
